# Comparison of Various Antimicrobial Agents for Thermoplastic Polymeric Retainers

**DOI:** 10.3390/polym14183753

**Published:** 2022-09-08

**Authors:** Kanket Kiatwarawut, Jintakorn Kuvatanasuchati, Boonyanit Thaweboon, Irin Sirisoontorn

**Affiliations:** 1 Department of Clinical Dentistry, Walailak University International College of Dentistry (WUICD), 87 Ranong 2 Road, Dusit, Bangkok 10300, Thailand; 2 Department of Oral Health Science, Walailak University International College of Dentistry (WUICD), 87 Ranong 2 Road, Dusit, Bangkok 10300, Thailand

**Keywords:** cleaning, clear retainer, disinfection, Essix, thermoplastic, Vivera

## Abstract

The thermoplastic retainers indicated a rising incidence of cariogenic bacteria such as *Streptococcus mutans.* A report suggested the case of a patient with severe gingival inflammation and dental caries as a result of inadequate appliance cleaning. This study aims to compare the various antimicrobial agents for thermoplastic polymeric retainers. A minimum bactericidal concentration (MBC) of acetic acid was determined. *Streptococcus mutans* biofilm was formed on punched 4-mm copolyester (Essix ACE^®^) and polyurethane (Vivera^®^) retainers after they were submerged in 0.12% chlorhexidine (CHX group), acetic acid (AA group), Polident Denture Cleanser^®^ (PD group), and Polident Pro Guard & Retainer^®^ (PR group). A crystal violet (CV) test was performed. The biofilm imaging was assessed by confocal laser scanning microscopy (CLSM). The results showed that all chemical disinfectants exhibited statistically significant differences (*p* < 0.05) compared to the positive control. This novel finding elucidated that 0.625% acetic acid is effective for antimicrobial in both copolyester and polyurethane retainers. However, only the CHX, PD, and PR groups could reduce biofilm mass. In addition, the CV assay cannot provide information about the actual number of living and dead bacteria. Furthermore, the LIVE/DEAD *Bac*Light assay was able to show the bacterial viability.

## 1. Introduction

A gold standard for cleaning thermoplastic polymeric retainers has never been published [1]. A study found that cariogenic bacteria such as *Streptococcus mutans* and *Lactobacillus* spp. are becoming more common in thermoplastic polymeric retainers [2]. According to Alshatti H. et al., the incidence and severity of white spot lesions were comparable among clear aligners, self-ligating brackets, and conventional brackets [3]. Another report suggested the case of a patient with severe gingival inflammation and incisal-edge and cusp-tip decay as a result of no appliance cleaning while having been eating and drinking for four consecutive months [4]. Wearing full-time retainers is an indisputably critical phase, as maintaining the final orthodontic outcome is one of the most important goals after treatment completion. Given that approximately 70% of orthodontic treatments result in retention failure [5] therefore retainers should be worn consistently.

The Essix ACE^®^ retainer, which is made primarily of copolyester, is both transparent and long-lasting. It was recently launched by Dentsply International to address deterioration and appearance issues with polypropylene polymer (Essix C+^®^) and polyethylene copolymer (Essix A+^®^) [6]. Align Technology recently introduced Vivera^®^ polyurethane retainers, which use the same 3D digital imaging cast fabrication technology as Invisalign^®^ aligners and smart track material with high elasticity and shape memory [7]. Studies show that copolyester-based and polyurethane-based retainers have recently become popular because of their clear and thin surfaces [8,9], and efficacy in keeping incisor position and alignment [10]. As far as we know, there is still no consensus on the comparison between tooth-relapse prevention and the characteristics modified after long-term disinfection of these retainers, which has led to an increase in studies on their properties and product care.

The physical surface of a target material plays a crucial role in bacterial adhesion among a variety of influential factors [7,11], including surface charge [12], hydrophobicity of the surfaces [13], and surface roughness on bacteria colonization [14]. Thermal plastics are hydrophobic polymers with positive surface charges. While *Streptococcus mutans* is a hydrophobic species, bacteria in an aqueous suspension may have a negative charge, increasing the likelihood of *Streptococcus mutans* adhering to thermoplastic surfaces and forming a biofilm.

Several agents, including chlorhexidine (CHX), cleaning tablets, and vinegar, are used to remove biofilm from the retainer. Chlorhexidine is an antimicrobial that works against a wide range of pathogens, including *Streptococcus mutans*, *Escherichia coli, Streptococcus sanguinis*, *Candida albicans*, and Staphylococcus aureus that is resistant to methicillin [15]. *Streptococcus mutans* was found to be significantly less prevalent in orthodontic retainers disinfected with chlorhexidine mouthwash [16,17]. Both orthodontics and prosthodontics have seen an increase in the use of these ready-to-use cleaning tablets. The efficiency of cleaning tablets in reducing bacteria adherence on thermoplastic sheets was demonstrated in an in vitro experiment in 2019 compared to the control [18]. However, a randomized clinical trial showed an insignificant difference in the bacterial count when the Essix^®^ was cleaned with various cleansing tablets compared with mechanical cleaning [19]. Due to its low cost, easy access, and antibacterial properties, several researchers have tried to use vinegar as a cleaning chemical agent for orthodontic appliances [20,21,22]. However, the optimal concentration of vinegar for retainer cleaning has never been established. Their modes of action and usage directions are distinct, and that requires further research [1].

This study revealed the uniqueness of different antibacterial agents for thermoplastic polymeric retainers using the biofilm quantification assay and LIVE/DEAD™ *Bac*Light™ fluorescent stain with confocal laser scanning microscopy.

## 2. Materials and Methods

This study evaluated the effects of 0.12% CHX (C-20 Chlorhexidine Antiseptic Mouth Wash^®^, Osoth Inter Laboratories, Bangkok, Thailand), acetic acid (AA), and two types of cleaning tablets (Polident Denture Cleanser^®^, GlaxoSmithKline PLC, Ermington, Australia (PD)), and Polident Pro Guard & Retainer^®^, GlaxoSmithKline PLC, Ermington, Australia (PR)) on *Streptococcus mutans* ATCC 25175 (ATCC, Manassas, VA, USA) biofilms.

The MIC and MCB of acetic acid were initially evaluated in the research. *Streptococcus mutans* was cultured in Tryptic Soy Broth (TSB). The bacterial suspension had to be diluted until it reached the 0.5 MacFarland standard threshold, or around 1.5 × 10^8^ CFU/mL. A 5% AA broth dilution (Carlo Erba^TM^, Milan, Italy) was then prepared. The final AA concentrations are 5%, 2.5%, 1.25%, 0.625%, and 0.3125%. The MBC of AA was used for the next step.

The thermoplastic sheets, Essix ACE^®^ (Dentsply International Inc., Charlotte, NC, USA) and Vivera^®^ (Align Technology Inc., Tempe, AZ, USA), were prepared to a diameter of 4 mm. In total, there are six groups of each plastic sheet: negative (Neg) control, positive (Pos) control, CHX, AA, PD, and PR. Each group contains seven thermoplastic specimens. They were disinfected for 20 min using ultraviolet light, then flipped over for another 20-min disinfection of the other side [23]. To coat the specimens, a pool of donated saliva was centrifuged with 5500× *g* for 10 min at 4 °C before collecting the top supernatant solution. The suspension was filtered through a 0.22 µm filter. The samples were submerged in the filter-sterilized suspension for two hours before testing. After being coated with saliva, only Pos, CHX, AA, PD, and PR specimens were put in a diluted *Streptococcus mutans* suspension (0.5 McFarland standard) and incubated at 37 °C for 48 h.

A PBS buffer (pH 7.2) was used to wash biofilm-forming specimens. The negative and positive groups were disinfected with sterile water, while the others were disinfected for 15 min with 0.12% CHX and AA (the concentration was equal to the MBC of AA). PD and PR disinfection times were 5 min according to the brands’ recommendation.

After disinfection, the researcher selected 6 treated specimens per group. They were washed three times in PBS before being fixed in 95% ethanol. Afterward, the specimens were stained with 0.1% CV, washed with sterile water to remove any overstained color, and allowed to dry completely.

The samples in each group were immersed in 100 µL of 33% acetic acid for 10 min in a 96-well microplate to dissolve the CV stain (1 specimen per well). The optical density at 595 nm was measured using the microplate reader (Infinite F50 plus, Tecan, Zürich, Switzerland). The experiment cycle was repeated four times to reduce human errors.

Microscopy and quantitative assays were performed using the LIVE/DEAD™ *Bac*Light™ Bacterial Viability Kit (Molecular Probe Inc., Eugene, OR, USA). The researcher applied a fluorescent stain working solution by adding 3 µL of SYTO 9 stain and 3 µL of propidium iodide (PI) stain to 1 mL of filter sterilized water. Without a CV assay, one sample from each group of agents was picked based on the results of the last test. Before being immersed in the staining solution, the treated specimens were rinsed three times with sterile water. For 15 min, the specimens were held at room temperature and were not exposed to light. The specimens were rinsed three times with filtered sterile water before being scanned with an Olympus Fluoview FV3000 Confocal Laser Scanning Microscope (Olympus Corporation, Tokyo, Japan).

### Statistical Analysis

The Kolmogorov-Smirnov test was used to estimate the normal distribution of data with SPSS Statistics 26 (IBM, Armonk, NY, USA). The data was examined using the Kruskal-Wallis test to compare the results of different agents. After that, Bonferroni’s test was used for post hoc analysis. In addition, the Mann-Whitney U test was used to compare biofilm removal between Essix ACE^®^ and Vivera^®^.

## 3. Results

The MIC and MBC of acetic acid against *Streptococcus mutans* were 0.312% and 0.625%, respectively. The MBC concentration from this step was used for the next experiment.

According to Stepanovic et al.’s modified Christensen adherence capability, published in 2000 [24], the mean OD of negative control of Essix^®^ specimens is 0.0345, with a standard deviation of 0.0024. So, the two numbers mentioned above were used to figure out the cut-off OD (ODc), which is equal to [0.0345 + 3 × 0.0024] = 0.0417.

Following this formula, the ODc of Vivera^®^ specimens is 0.0664 (mean OD of Vivera^®^ negative control is 0.0454, and the standard deviation equals 0.0070). The adherence level classification is shown in Table 1 below.

Table 2 shows the results of comparing the mean OD of agents in Essix^®^ and Vivera^®^ specimens using the above formula.

After analyzing each group, all chemical disinfectants were statistically distinct from the positive control, indicating that all agents had bactericidal effects on the Essix^®^ specimens. Furthermore, the biofilm removal degree was considerably higher in the CHX and PR groups based on the evaluation of each agent, as shown in Table 3.

The results of the Vivera^®^ specimens were similar to those of the Essix^®^ specimens; all chemical disinfectants exhibited significant differences compared to the positive control, meaning all agents contained bactericidal properties. After evaluating each agent, the results showed that the CHX, PD, and PR groups could remove much more biofilm than the AA groups, as shown in Table 4.

**Figure 1:**
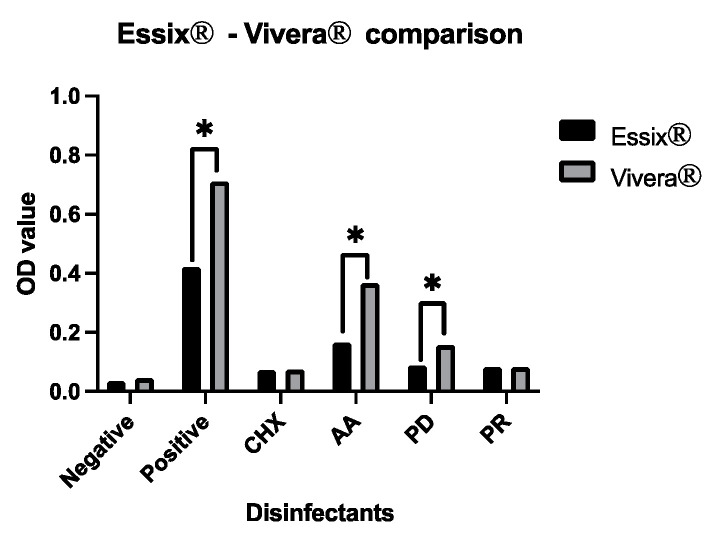
The comparison between two types of thermoplastic retainer after cleaning with each agent, * (*p* < 0.05).

The effects of each chemical (Pos, AA, and PD) on the two types of plastics (Essix^®^ and Vivera^®^) were noticeably different, as shown in Figure 1.

**Figure 2:**
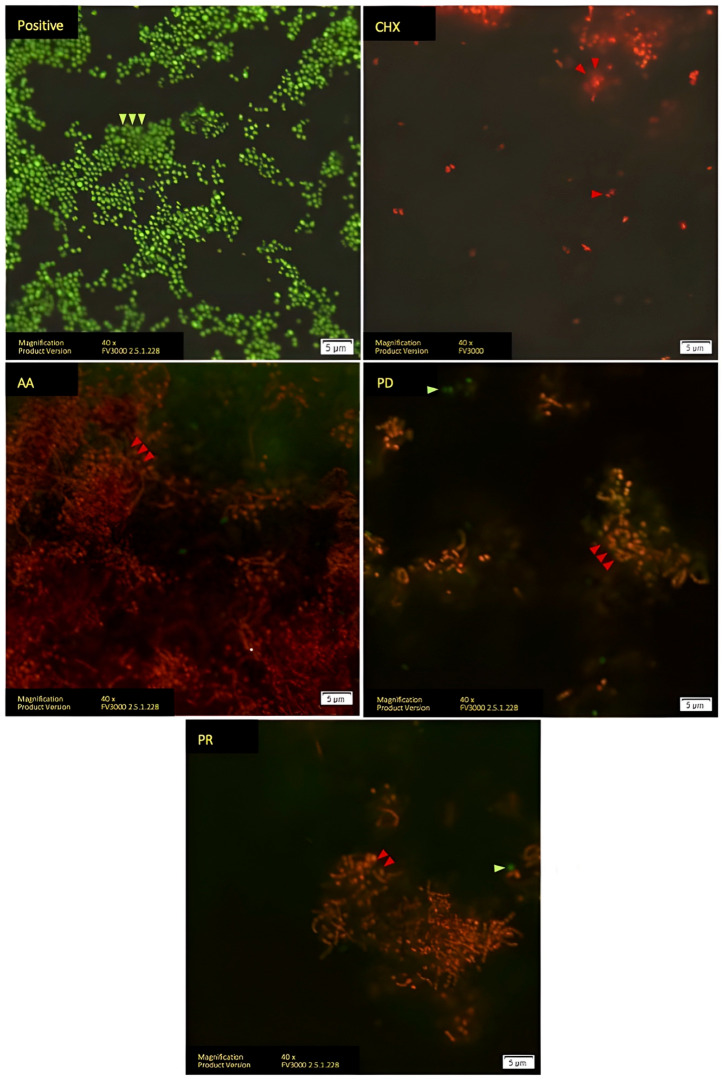
LIVE/DEAD staining analyzed with confocal microscope of Essix^®^ retainer. Living cells stained with SYTO9 are indicated with green arrows. Dead cells stained with PI are indicated with red arrows.

As assessed by Confocal Laser Scanning Microscopy (CLSM), the biofilms on Essix^®^ differed between groups. The living cells are presented in green, but the dead cells are shown in orange or red. Positive groups are mainly displayed in green because sterile water has no antimicrobial effect. However, CHX, AA, PD, and PR groups were mainly in orange or red, which clarifies the antibacterial activity of these agents. Aligned with the results of the CV assay, the positive and AA groups exhibited higher densities compared with the other groups. Hence, as can be shown in Figure 2, CHX, PD, and PR all have qualities that can reduce biofilm, but AA does not.

**Figure 3:**
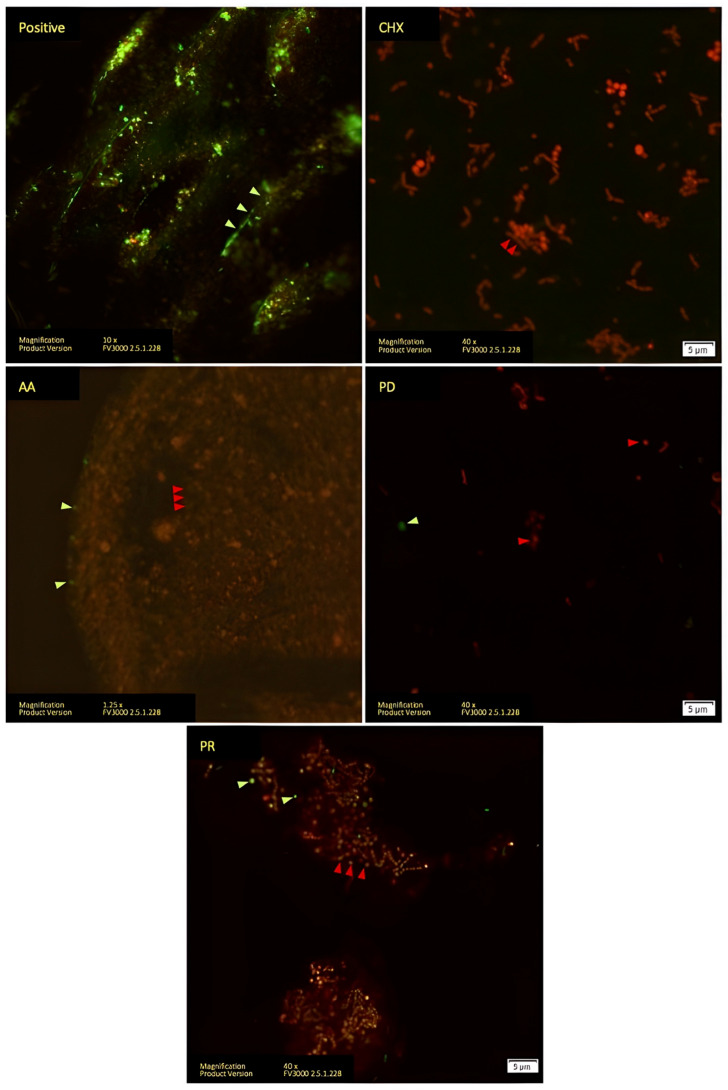
LIVE/DEAD staining analyzed with confocal microscope of Vivera^®^ retainer. Living cells stained with SYTO9 are indicated with green arrows. Dead cells stained with PI are indicated with red arrows.

The results were similar to that of Vivera^®^, where the CHX, AA, PD, and PR groups primarily exhibited in red, but the AA group had highly populated bacteria. The PD and PR groups were effective in disinfection and biofilm mass eradication. The novel aspect of the positive group was discovered at a low magnification (10X). Bacterial adherence was identified in the surface’s niche, which may make disinfectant penetration more difficult according to the fingerprint pattern of the Vivera^®^ surface design, as shown in Figure 3.

**Figure 4:**
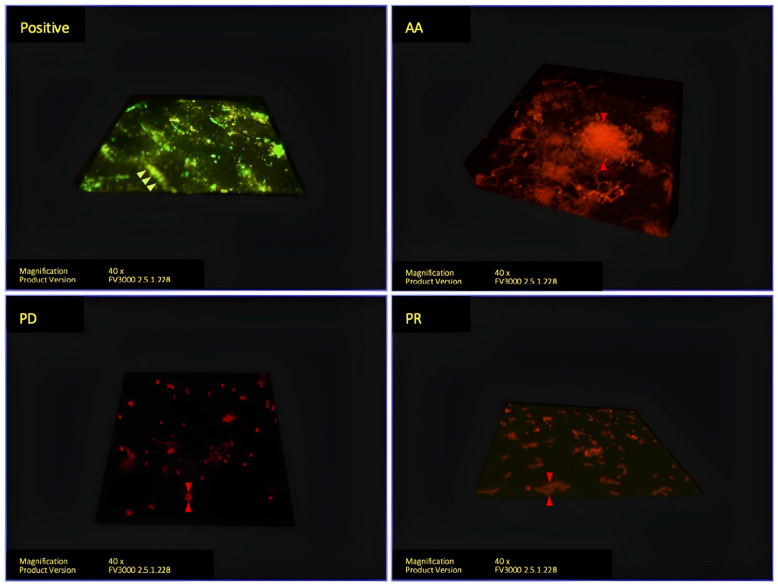
Vivera^®^ retainer 3D imaging analyzed with confocal microscope. Living cells stained with SYTO9 are indicated with green arrows. Dead cells stained with PI are indicated with red arrows.

From Figure 4, a 3D diagram of tested chemicals, the corrugated pattern of the Vivera^®^ retainer was still seen in the positive group. Furthermore, the difference in the biofilm thickness after disinfecting with various chemicals was detected. The biofilms of the PD and PR groups were similar in that they were thin and widespread in biofilm size, but the AA biofilm was thicker and denser.

## 4. Discussion

Copolyesters and polyurethane have recently become interesting materials because of their potential biodegradability due to their hydrolysable ester bonds [25,26,27]. This copolyester contains aliphatic polyester and terephthalic acid [27] which are considered to be susceptible to microbial attack. Aliphatic polyester breaks down in two steps: depolymerization, or surface erosion, and enzymatic hydrolysis, which makes water-soluble intermediates that microorganisms can use [28]. Polyurethane, such as copolyesters, is a biodegradable substance because the urethane bond in polyurethane has been reported to be susceptible to microbial attack. The hydrolysis of polyurethane ester bonds is thought to be the mechanism of polyurethane biodegradation [29]. However, investigations involving oral microbes are uncommon.

In this investigation, the researcher applied an initial concentration of 5% acetic acid because the AA content of vinegar commercially available was approximately 5%. There are only a few reports on the concentration suitable for application as a disinfectant. Despite the fact that the higher concentration will have a higher disinfecting effect, the pungent odor on the retainer remains a problem. So, the first step of the investigation was to find the MIC and MBC. The concentration used was 0.625%.

As represented by the OD value, biofilm removal by each agent was distinct, partly due to their distinct mechanisms. For CHX to kill bacteria, the germs must be bound to cationic molecules with a negative charge [30]. The agent entered the bacterial cytoplasm through passive diffusion before attacking the bacterial cytoplasmic or inner membrane or plasma membrane [16]. Bacterial cells die when the semipermeable membrane is damaged, enabling intracellular organs to leak out [31]. Huge quantities of intracellular components coagulated when chlorhexidine was in a higher concentration [32]. As a result, the cytoplasm becomes solid, with a consequent reduction in leakage [18]. Nevertheless, a study showed the same efficacy of 0.12% or 2.0% CHX solutions for cleaning intraoral appliances [33].

CHX is not only bactericidal but also capable of biofilm eradication. A study in 2011 revealed that, after treatment with CHX [34], the biofilm mass could be reshaped and reduced. Furthermore, in the presence of CHX, the level of Extracellular Polymetric Substances (EPS), the protective barrier for mutated biofilm, decreased by nearly 70% [35].

When bacteria are exposed to low-acidity acids, they are more susceptible than they would otherwise be, as universally acknowledged. Weak acids may permeate bacterial membranes more easily than strong acids due to the unbalance between inner and outer proton concentrations. The non-ionized forms engage with periplasmic protons pumped out by F1F0 ATPase and can diffuse freely across hydrophobic membranes [36,37]. Acid-induced protein unfolding and membrane damage, including DNA, may occur if the internal pH of the cell (typically 7.6 [38,39] in neutralophilic bacteria) is higher than the pH of the external acid solution (typically around pH 5.8 [40]).

For cleaning tablets, there are many effective bactericidal ingredients such as citric acid, sodium carbonate, potassium monopersulfate, and sodium carbonate peroxide. Chemical soak-type products, such as effervescent tablets, break down quickly in water to generate an alkaline peroxide solution in which sodium perborate is dissolved. Due to this peroxide solution’s eventual release of oxygen, chemical and mechanical cleaning are achievable [41]. The mechanical effect of the effervescent tablet aids in the reduction of biofilm mass. Even though PR is a new product made just for retainers, its ingredients are the same as those in PD.

This study reported that CHX, PD, and PR could kill microorganisms and remove biofilm. However, AA can only kill bacteria, implying that additional mechanical cleaning is still necessary. For instance, brushing on the retainer surface before or after immersion in disinfectant is recommended [42], particularly for Vivera^®^, where adhesion is typically observed, as shown in Figure 1. In addition, the surface of the Vivera^®^ retainers had depressions and flaws that could serve as breeding grounds for germs [43].

However, either chemical or mechanical cleaning requires careful application, as a study found that long-term use of chemicals for cleaning, such as vinegar, Polident^®^, and mouthwash, changes light transmittance and flexural modulus, which can damage the appearance and durability of both copolyester and polyurethane retainers [20,21]. At any rate, even a general oral application of the clear retainer is reported to trigger discoloration [44]. Nevertheless, the thermoplastic polymeric retainer tends to discolor and crack after 6 to 9 months, requiring replacement [45].

According to the CV assay, if research is required to evaluate the antimicrobial activity of disinfectants, merely using a simple CV assay may not be sufficient and may lead to misinterpretation since CV binds indifferently to negatively charged live and dead bacteria as well as EPS polysaccharides, which may also cause overestimation.

The LIVE/DEAD *Bac*Light assay is another possibility to monitor both the killing of bacteria and the removal of biofilm. The core concept is that the green fluorescent (SYTO9) dye can permeate all bacterial membranes and bind DNA. In the second dye with red-fluorescent PI, only damaged bacterial membranes are permeable. These advantages can present the antibacterial activity of various disinfectants. Furthermore, the biofilm mass reduction can be detected, which conforms to the CV assay. However, CLSM could only display specific sections, and the captured areas where no specimen is present could affect the interpretation of the results. Moreover, due to the inability to count all bacterial cells, this method only yields semiquantitative results [46,47].

## 5. Limitations

In the actual oral cavity, the microorganisms are multispecies. They present the interaction across species, resulting in enhanced resistance and virulence to antimicrobial agents. However, this study selected only *Streptococcus mutans,* a cariogenic bacteria, which might not generalize to the real situation. Moreover, the study was unable to determine the optimal frequency of cleaning retainers with chemicals, nor did it examine the long-term impact on changes in the material’s physical properties, such as paint adhesion or aging.

## 6. Conclusions

0.625% AA is effective as an antibacterial on both copolyester and polyurethane. In addition, CHX, PD, PR, and AA were tested to be effective at killing bacteria. However, only the CHX, PD, and PR could reduce biofilm mass. In addition, the CV assay was able to present the amount of biofilm mass, but it could not provide information about the actual number of living and dead bacteria. Moreover, the LIVE/DEAD *Bac*Light assay with confocal microscopy demonstrated bacterial vitality in semiquantitative results.

## Figures and Tables

**Table 1:** The classification of bacterial adherence on Essix^®^ and Vivera^®^ specimens.

Bacterial adherence classification of Essix^®^ specimens					
OD	≤	0.0417			Non-adherent (−)
0.0417	<	OD	≤	0.0833	Weakly adhearent (+)
0.0883	<	OD	≤	0.1668	Moderately adhearent (++)
0.1668	<	OD			Strongly adhearent (+++)
**Bacterial adherence classification of Vivera^®^ specimens**					
OD	≤	0.0664			Non-adherent (−)
0.0664	<	OD	≤	0.1328	Weakly adhearent (+)
0.1328	<	OD	≤	0.2656	Moderately adhearent (++)
0.2656	<	OD			Strongly adhearent (+++)

**Table 2:** The level of bacterial adherence on Essix^®^ retainer after cleaning.

Group (Essix^®^)	Mean OD	Level	Group (Vivera^®^)	Mean OD	Level
Neg	0.0345	-	Neg	0.0454	-
Pos	0.4295	+++	Pos	0.7799	+++
CHX	0.0672	+	CHX	0.0817	+
AA	0.1626	++	AA	0.3736	+++
PD	0.0830	+	PD	0.1649	++
PR	0.0834	+	PR	0.1086	+

**Table 3:** Pairwise comparison table of antimicrobial activity on Essix^®^ specimens.

Agent	Neg	Pos	CHX	AA	PD	PR
Neg	1.000	0.000 ^a^	0.010 ^a^	0.000 ^a^	0.000 ^a^	0.000 ^a^
Pos	0.000 ^a^	1.000	0.000 ^a^	0.027 ^a^	0.000 ^a^	0.000 ^a^
CHX	0.010 ^a^	0.000 ^a^	1.000	0.000 ^a^	0.048 ^a^	0.077
AA	0.000 ^a^	0.027 ^a^	0.000 ^a^	1.000	0.007 ^a^	0.005 ^a^
PD	0.000 ^a^	0.000 ^a^	0.048 ^a^	0.007 ^a^	1.000	0.847
PR	0.000 ^a^	0.000 ^a^	0.077	0.005 ^a^	0.847	1.000

^a^ (*p* < 0.05).

**Table 4:** Pairwise comparison table of antimicrobial activity on Vivera^®^ specimens.

Agent	Neg	Pos	CHX	AA	PD	PR
Neg	1.000	0.000 ^a^	0.004 ^a^	0.000 ^a^	0.000 ^a^	0.000 ^a^
Pos	0.000 ^a^	1.000	0.000 ^a^	0.107	0.000 ^a^	0.000 ^a^
CHX	0.004 ^a^	0.000 ^a^	1.000	0.000 ^a^	0.095	0.449
AA	0.000 ^a^	0.107	0.000 ^a^	1.000	0.021 ^a^	0.001 ^a^
PD	0.000 ^a^	0.000 ^a^	0.095	0.021 ^a^	1.000	0.353
PR	0.000 ^a^	0.000 ^a^	0.449	0.001 ^a^	0.353	1.000

^a^ (*p* < 0.05).

## Data Availability

Not applicable.
